# Unrealistic Optimism and Risk for COVID-19 Disease

**DOI:** 10.3389/fpsyg.2021.647461

**Published:** 2021-06-04

**Authors:** Jeffrey Gassen, Tomasz J. Nowak, Alexandria D. Henderson, Sally P. Weaver, Erich J. Baker, Michael P. Muehlenbein

**Affiliations:** ^1^Department of Anthropology, Baylor University, Waco, TX, United States; ^2^Waco Family Medicine, Waco, TX, United States; ^3^Department of Computer Science, Baylor University, Waco, TX, United States

**Keywords:** unrealistic optimism, risk perception, COVID-19, SARS–CoV−2, optimism bias, pre-existing condition

## Abstract

Risk perception and consequently engagement in behaviors to avoid illness often do not match actual risk of infection, morbidity, and mortality. Unrealistic optimism occurs when individuals falsely believe that their personal outcomes will be more favorable than others' in the same risk category. Natural selection could favor overconfidence if its benefits, such as psychological resilience, outweigh its costs. However, just because optimism biases may have offered fitness advantages in our evolutionary past does not mean that they are always optimal. The current project examined relationships among personal risk for severe COVID-19, risk perceptions, and preventative behaviors. We predicted that those with higher risk of severe COVID-19 would exhibit unrealistic optimism and behave in ways inconsistent with their elevated risk of morbidity and mortality. Clinical risk scores for severe COVID-19 were calculated and compared with COVID-19 threat appraisal, compliance with shelter-in-place orders (March 13–May 22, 2020) and travel restrictions, compliance with public health recommendations, and potential covariates like self-rated knowledge about COVID-19 in a robust dataset including 492 participants from McLennan County, TX, USA. While those with high clinical risk acknowledged their greater likelihood of experiencing severe illness if infected, they actually reported lower perceived likelihood of becoming infected in the first place. While it is possible that those with higher clinical risk scores truly are less likely to become infected, the pattern and significance of these results held after controlling for possible occupational exposure, household size, and other factors related to infection probability. Higher clinical risk also predicted more recent travel within Texas and lower distress during the pandemic (i.e., feeling less stressed, depressed, and helpless). Additional behavioral data suggested that those with higher clinical risk scores did not generally behave differently than those with lower scores during the shelter-in-place order. While unrealistic optimism may provide some short-term psychological benefits, it could be dangerous due to improper assessment of hazardous situations; inferring that optimism bias has evolutionary origins does not mean that unrealistic optimism is “optimal” in every situation. This may be especially true when individuals face novel sources (or scales) of risk, such as a global pandemic.

## Introduction

The spread of the SARS CoV-2 virus since late 2019 has generated a public health crisis, creating economic uncertainties (Pak et al., 2020), interrupting well-established food supply chains (Rizou et al., 2020), and ultimately resulting in large scale hospitalizations and deaths (Meyerowitz-Katz and Merone, 2020; Reese et al., 2020; Weinberger et al., 2020). The SARS-CoV-2 virus transmitted rapidly around the globe, resulting in millions of cases of COVID-19 disease. Although this serious and persistent threat remains, individuals' perceptions of risk and, consequently, their engagements in behaviors to avoid illness (e.g., wearing face coverings, social distancing, hygiene) have often not matched their own actual degree of risk of infection, morbidity, and mortality. For example, many people refuse to wear face coverings, even within healthcare facilities (Lehmann and Lehmann, 2020). Others continue to attend large gatherings, despite findings that these events confer considerable risk of SARS-CoV-2 virus exposure (Ebrahim and Memish, 2020; Majra et al., 2020; Sassano et al., 2020).

In the present project, we sought to build on these findings by examining relationships among individuals' personal risk for severe COVID-19 disease, risk perceptions, and preventative behaviors in a large community cohort. Specifically, we calculated clinical risk scores for severe COVID-19 disease and compared them to individuals' own perceptions of their risk. Further, we examined whether clinical risk scores were related to compliance with shelter-in-place orders, travel restrictions, and public health recommendations, as well as reported distress during the pandemic. Combining insights from the evolutionary and cognitive sciences, we predicted that those with a high clinical risk for severe COVID-19 disease would exhibit unrealistic optimism, characterized by an underestimation of their personal vulnerability and behaving in a manner inconsistent with their elevated risk of morbidity and mortality.

### Unrealistic Optimism

Personal risk reduction relies on factors such as belief about the likelihood of an adverse event taking place and belief about the severity of that event (Rippetoe and Rogers, 1987; Floyd et al., 2000; Milne et al., 2000). Estimation of the probability and severity of a noxious event is important for assessing the relative costs and benefits of taking steps to decrease the event's likelihood. For example, underestimating one's risk may result in failure to prevent an avoidable negative outcome. On the other hand, overestimating one's risk may yield opportunity costs, that is, probable gains lost in the process of risk mitigation.

Despite these potential costs associated with inaccurate risk assessment, many people experience positive illusions regarding their individual risk in which they underestimate their own likelihood of experiencing negative outcomes (McKay, 2009). This tendency for individuals to falsely believe that their personal outcomes will likely be more favorable than others' in the same risk category is called “optimism bias” or “unrealistic optimism” (Sharot, 2011; Shepperd et al., 2015; Jefferson et al., 2017). Given that these terms are often used interchangeably in the literature, we henceforth refer to this bias as unrealistic optimism (Jefferson et al., 2017). Research has found that unrealistically optimistic beliefs are defined by their stability, persisting through selective attention for new information that confirms positive beliefs while disregarding information that contradicts the beliefs (Sharot et al., 2011). Moreover, these beliefs are genuinely accepted as truth by the individual (Jefferson et al., 2017).

The phenomenon of unrealistic optimism is widespread, applying to a variety of situations from health to stock market trading (Makridakis and Moleskis, 2015; Reyes-Velázquez and Sealey-Potts, 2015). Examples include an individual's beliefs that they are more likely than other players to win when rolling dice at a casino, or a married couple's tendency to underestimate the probability that their marriage will end in divorce relative to other couples (Jefferson et al., 2017). Even non-human animals, such as European starlings and mice, exhibit unrealistic optimism in certain choice tasks (Harding et al., 2004; Matheson et al., 2008).

### Evolution and Unrealistic Optimism

Why is unrealistic optimism so common? It is difficult to imagine that underestimating risk, a potential opponent, or the difficulty of a task would be beneficial or evolutionarily adaptive. However, unrealistic optimism remains entrenched across human populations. A wide body of research has found that optimism, more generally, is linked to many positive health outcomes. For example, optimism is associated with a lower prevalence of high blood pressure across multiple populations (Räikkönen et al., 1999; Räikkönen and Matthews, 2008). Further, a recent meta-analysis identified a connection between optimism and both lower risk of cardiovascular events and all-cause mortality (Rozanski et al., 2019).

Regarding unrealistic optimism more specifically, insights from the evolutionary sciences indicate that selection could favor overconfidence as long as the benefits of unrealistic optimism outweigh its costs. Specifically, game theory models reveal that overconfidence can emerge as an evolutionarily stable strategy across a wide range of environments, and should be strongest under increasingly uncertain conditions (Johnson and Fowler, 2011). Unbiased risk estimation, on the other hand, is predicted by this model to be stable only under a limited set of specific conditions (Johnson and Fowler, 2011). These findings suggest that unrealistic optimism confers tangible benefits that favor its selection, particularly in environments characterized by risk and uncertainty.

Error Management Theory (EMT), an evolutionary framework for understanding cognitive biases (Nettle, 2004; Haselton and Nettle, 2006; McKay, 2009; Johnson and Fowler, 2011), provides key insights into the benefits of unrealistic optimism under uncertainty. Due to volatility in the environment and constraints on perception, totally accurate assessment of threat, and prediction of outcomes related to that threat, are often difficult or impossible. Under uncertainty, errors in judgment usually fall into one of two categories: false positives (i.e., assuming a threat exists when it does not) or false negatives (i.e., assuming a threat does not exist when it does). EMT posits that selection will favor bias toward the less costly type of error (Haselton and Buss, 2000; Haselton and Nettle, 2006). An analogy often used to describe such patterns of error management is the smoke detector (Nesse, 2001; Haselton and Nettle, 2006). Of course, it would be best to have a very accurate smoke detector that can perfectly distinguish between burned toast and actual fire in an apartment. However, errors are unavoidable, and it is imperative that the smoke detector always senses a real fire. Accordingly, the smoke detector alarm, from time to time, will go off when we burn toast (false positive), but it rarely fails to go off in the event of an actual fire (false negative).

This framework may also help explain unrealistic optimism. Selection could favor biases toward “optimistic error” if true probabilities of success are incompletely known, and erring on the side of optimism provides greater benefits and/or bears fewer costs than erring on the side of pessimism (Haselton and Nettle, 2006; Jefferson, 2017; Jefferson et al., 2017). For example, due to males' higher reproductive variance relative to females' (i.e., in sexually-reproducing species with greater obligate female investment in offspring), the costs associated with missing out on mating opportunities are especially high for males, exceeding the costs of wasting energy on unsuccessful mating pursuits (Trivers, 1972; Alcock, 1993). EMT then proposes that males should be more prone to false positive, rather than false negative, errors when searching for mating opportunities. In other words, men should be unrealistically optimistic about women's interest in them. Consistent with this prediction, research has found that men, but not women, tend to overperceive cues of interest from the opposite sex (Haselton, 2003).

More generally, studies have found that positive illusions—such as unrealistic optimism—also yield psychological benefits in the face of risky or uncertain situations (Taylor and Brown, 1994; McKay, 2009; Makridakis and Moleskis, 2015), increasing motivation and promoting resilience in response to adversity (Bénabou and Tirole, 2002; Johnson and Fowler, 2011; Kleiman et al., 2017). For example, research has found that individuals who are unrealistically optimistic about how positively they will feel in the future are better able to handle stress (Colombo et al., 2020). Others have shown that college students with more optimistic expectations for their academic performance invest more quality effort into studying and are more satisfied with their decision-making (Lench et al., 2021). In sum, amid uncertainty, unrealistic optimism may yield benefits in the form of promoting resilience and motivating adaptive behaviors (McKay, 2009).

### Maladaptive Optimism

While EMT provides a useful framework for understanding optimistic errors, there are arguments against evolutionary accounts of unrealistic optimism. Jefferson (2017) presents three potential problems with reducing unrealistic optimism to the outcome of an evolutionary cost-benefit analysis as is done in EMT. First, unrealistic optimism about health risks may lead to behaviors that contribute to morbidity and mortality. It is difficult to imagine that such costs are outweighed by the costs of assuming the worst in such scenarios. For example, one study found that in individuals with type II diabetes, greater optimism regarding future heart attack risk was actually associated with a higher incidence of lifestyle factors (e.g., smoking) that increase the likelihood of cardiovascular disease (Karl et al., 2020). A separate study found that smokers who were unrealistically optimistic about their lung cancer risk were less likely to have a smoking cessation plan than those who were less optimistic (Dillard et al., 2006).

Second, unrealistic optimism may lead individuals to believe that a positive result will occur even if they do not take action. In other words, unrealistic optimism might lead to complacency (Jefferson, 2017). Consistent with this possibility, a survey of 100 college students found that of 45 different health- and life-threatening events, participants showed an optimism bias for 34 of them. Further, unrealistically optimistic evaluations of risk from these hazards were associated with reduced worry about the occurring events (Weinstein, 1982), suggesting that overconfidence about one's likelihood of experiencing health problems may reduce motivation to take the steps necessary to mitigate risk. Many have even blamed unrealistic optimism for inadequate preparation for natural disasters or financial bubbles (Johnson and Levin, 2009; Johnson and Fowler, 2011; Shepperd et al., 2015; Glöckner, 2016; Michailova and Schmidt, 2016).

Lastly, Jefferson (2017) suggests that there exist many proximate moderators of unrealistically optimistic tendencies that are not accounted for by EMT. While the existence of these moderators does not necessarily contradict EMT, it suggests that factors other than the cost-benefit ratio of false positives and false negatives also influence optimism. Namely, better access to information, concerns about accountability, and mood states each affect optimistic beliefs (Sweeny et al., 2006). Perhaps an even more troubling moderating factor is that it is often those at the highest risk for adverse outcomes who are most unrealistic about their circumstances (Ferrer et al., 2012; Morgan et al., 2019; Dolinski et al., 2020). For instance, one study found that individuals who were more optimistic about their future risk for heart disease actually had greater intima-media thickness, an early marker of atherosclerosis (Ferrer et al., 2012). Others found that young people tend to underestimate their risk of household accidents relative to their older counterparts, despite being much more likely to experience these accidents (Morgan et al., 2019).

A similar pattern has been observed in the context of the current pandemic. For example, one study found that men, in particular, were unrealistically optimistic about their likelihood of SARS-CoV-2 infection (Dolinski et al., 2020), despite having a higher risk of infection and mortality from COVID-19 than women (Chakravarty et al., 2020). This is of grave concern because unrealistic optimism about one's likelihood of infection from the SARS-CoV-2 virus or severe illness from COVID-19 disease, may translate into dire consequences for those with high risk of infection, morbidity, and mortality worldwide, mainly because men are more likely to risk their health and disregard preventative measures. One potential explanation for this pattern of results is that women tend to have more compassionate attitudes and show higher dislike than men for others' suffering (Atari et al., 2020; Luoto and Varella, 2021). Women also tend to be higher than men in COVID-19-related disgust, as well as general risk aversion and neuroticism (Luoto and Varella, 2021).

These findings present an additional puzzle for evolutionary explanations of unrealistic optimism. Specifically, why do those more likely to experience an adverse outcome tend to underestimate their risk relative to those less likely? While there are a number of possible answers to this question, we discuss two here. First, considering this question within the EMT framework, it is possible that the costs of risk aversion are higher for those with a greater risk compared to a lesser risk in certain situations (Haselton and Buss, 2000). Specifically, the opportunity costs associated with pessimism might disproportionately affect those at higher risk if the conflict between avoiding risk and achieving other fitness-relevant goals is particularly strong for these individuals. For example, young adults (males in particular) often discount their risk of sexually transmitted infections (STIs) to a greater extent than older adults, despite being at a much higher risk (Ethier et al., 2003; Wolfers et al., 2011; Syme et al., 2017). This may be because the benefits of mating success, which are on average higher for younger adults than older adults, overshadow the costs associated with STIs. Such conflicts between adaptive domains (in this case, mating success and disease avoidance) constitute an adaptive metaproblem (Al-Shawaf, 2016; Rantala et al., 2019; Varella et al., 2021).

A second, related possibility is that high-risk individuals have a greater need for the aforementioned psychological benefits related to unrealistic optimism than low-risk individuals. That is, unrealistically optimistic beliefs may help those at an especially high risk for adverse events cope with the reality of their situation (Taylor and Brown, 1994; McKay, 2009; Makridakis and Moleskis, 2015). For example, with COVID-19 risk, in the absence of any optimistic buffer, those with a higher risk for morbidity and mortality would be expected to experience a great deal of distress compared to those with a lower risk. In addition to bearing psychological costs, such distress may also prevent high-risk individuals from meeting key affiliative (and other fitness-relevant) goals that are especially salient under threat (Dezecache et al., 2020; Varella et al., 2021). This conflict between affiliative needs and disease avoidance can, again, be thought of as representing an adaptive metaproblem (Al-Shawaf, 2016; Rantala et al., 2019; Varella et al., 2021). Furthermore, high-risk individuals may also be employing a “free-riding” strategy, enjoying the safety benefits of others' cautiousness while not adjusting their behavior commensurate with their high risk for morbidity and mortality (Yong and Choy, 2021). In doing so, these individuals might be able to achieve other fitness goals without drastically increasing their already elevated risk.

### Unrealistic Optimism and the COVID-19 Pandemic

While these insights may help to explain how natural selection could favor optimism biases, particularly in high-risk individuals, it is still unclear why unrealistic optimism would operate in contexts where it almost certainly increases the likelihood of serious adverse outcomes. One possibility is that unrealistic optimism leads to maladaptive outcomes, especially when individuals are faced with types (or scales) of risk that were not present in ancestral environments. For example, facing risk of cardiovascular disease—and being presented with knowledge about how to prevent it—is an evolutionarily novel situation. Accordingly, individuals may be poorly equipped to effectively weigh the costs (e.g., managing diet, exercising, etc.) and benefits (reduced odds of mortality) of taking steps to mitigate the risk of heart attacks and strokes, events that may not occur for many years into the future.

Although infectious diseases have been a threat throughout human history, the extremely high population density and international mobility that allowed the current large-scale pandemic to develop are novel. Accordingly, individuals may be unrealistically optimistic about their likelihood of infection from the SARS-CoV-2 virus or severe illness from COVID-19 disease, translating to dire consequences for those with high risk of infection, morbidity, and mortality worldwide. For example, the 2005 H5N1 avian influenza outbreak was a major public health challenge in many Asian, European, and African countries (de Zwart et al., 2007). Although this outbreak never reached pandemic levels, the highly pathogenic nature of this virus was significant (Peiris et al., 2007). In Hong Kong, where risk of exposure was elevated relative to many other countries, local residents perceived only moderate risk from buying live poultry. Specifically, only 36% of respondents in one survey agreed that purchasing live chickens was risky, and over 78% of households reported buying them during the peak of the epidemic in Asia (Fielding et al., 2005).

Moreover, while the personal health problems associated with COVID-19 are imminent, some people have still not experienced negative outcomes related to COVID-19 (or any other serious infectious disease outbreak) first-hand. In this way, the consequences of COVID-19 may seem, for some, distant in space in such a way that they do not perceive it as a significant threat. This may lead to them underestimating the risk of becoming infected or developing severe symptoms in general, tipping the scale toward unrealistic optimism and reducing investment in behaviors to reduce risk. In such a fashion, there was a false sense of security in certain countries and regions during the original SARS-CoV-1 epidemic (de Zwart et al., 2007). Education in the United States and Canada about the risks of SARS-CoV-1 virus was relatively unsuccessful at a large scale, as most of the populations remained unaware of the potential impacts of the virus (Blendon et al., 2004), in part because of its low level of spread (Blendon et al., 2004), and despite the fact that the United States and Canada experienced significant economic damage. In contrast, those in the Netherlands reported high levels of awareness for the SARS-CoV-1 virus (Brug et al., 2004). Although high perceptions of risk may have caused more worry, individuals also reported taking additional precautions to avoid the virus (Brug et al., 2004).

### Current Aims

The purpose of the present project was to examine relationships among personal risk for severe COVID-19 disease, risk perceptions, and preventative behaviors in a large community cohort. It was predicted that those with higher clinical risk of severe COVID-19 disease (as determined by self-reported pre-existing conditions, demographic factors, and clinical characteristics) would exhibit unrealistic optimism and behave in ways inconsistent with their elevated risk of morbidity and mortality. Consistent with previous research on unrealistic optimism (Weinstein, 1982; Reyes-Velázquez and Sealey-Potts, 2015; Shepperd et al., 2015; Jefferson et al., 2017), we predicted that this positive illusion may partially buffer high-risk individuals from the psychological consequences of the current pandemic. However, supporting the hypothesis that unrealistic optimism may lead to maladaptive outcomes when individuals face evolutionarily novel threats, we predicted that individuals with a higher risk of developing severe COVID-19 disease would not take more precautions than those with a lower risk.

## Methods

### Overview and Study Design

Data analyzed for the present study were collected as part of the Waco COVID Survey, a serological surveillance project of SARS-CoV-2 virus in McLennan County, Texas, United States. McLennan County comprises ~1,000 square miles in Central Texas, with a 2019 population estimate of 256,600 individuals with 27% Hispanic or Latina/Latino and 14.8% African American, 14.7% age 65 and above, 24.2% with a Bachelor degree or higher, 18% living below the national poverty line, and 18.4% without health insurance (while under the age of 65) (http://co.mclennan.tx.us/; https://www.census.gov/quickfacts/mclennancountytexas).

The primary purpose of this larger study was to prospectively determine the incidence of SARS-CoV-2 virus infection in several hundred asymptomatic individuals over the course of 4 months following the relaxation of shelter-in-place orders (shelter-in-place began on March 13th in Texas, with non-essential businesses opening back up on May 1st, 18th, and 22nd). This project was based on a repeated sampling protocol of targeted (non-random) clusters of individuals (all asymptomatic) that varied by exposure risk: those working in healthcare (including first responders), essential service employees (e.g., gas station attendants, lawn maintenance workers, grocery store employees, restaurant workers involved in food preparation for home delivery services or carry-out), employees in businesses and other organizations that reopened in May (e.g., restaurants, movie theaters, churches), and those who had claimed to strictly follow shelter-in-place and all public health recommendations. None of the participants included in the present analysis tested positive for IgG antibody against the SARS-CoV-2 virus (using EUROIMMUN COVID-19 ELISA with emergency use authorization).

Recruitment took place via Facebook, Twitter, and the Waco Tribune-Herald newspaper. Participation was limited to residents of McLennan County, Texas (since December 2019), age 18 years or older, fluency in English or Spanish (all materials were available in both languages), and absence of any signs or symptoms of COVID-19 disease, including cough, shortness of breath or difficulty breathing, pain or pressure in the chest, body temperature at or above 100 degrees Fahrenheit (37.8 degrees Celsius), chills, repeated shaking, sore throat, temporary loss of taste or smell, persistent headache, inability to stay awake, recent confusion, blush lips or face, muscle pain, vomiting, nausea, or diarrhea. Potential participants registered on a HIPAA-compliant website and then completed an extensive online questionnaire before visiting the Madison Cooper Community Clinic of Waco Family Medicine for anthropometric measurements and a venous blood draw. Participants made repeated visits monthly for venous blood draws to determine anti-SARS-CoV-2 IgG antibody levels and complete blood count. The data in the present analysis include those collected in the initial intake questionnaire.

The questionnaire included 103 questions (mostly multi-part each; ~30 min in length) about demographics, education, socioeconomic status, household composition (and health status of household members), religiosity, political leniency, occupation history, use of personal protective equipment, hygiene, compliance with shelter-in-place orders, use of face coverings, social distancing, travel, changes in behavior since the pandemic began, current and past health, medication usage, any pre-existing conditions, knowledge and attitudes regarding the SARS-CoV-2 virus and COVID-19 disease, diet, alcohol consumption, activity levels, sleep, general risk avoidance, mental health and stress, and general affect, among other questions. Specific questions used in the present analysis (besides demographics) are listed below.

In response to the public health emergency of the SARS-CoV-2 pandemic, this project functioned as a public health surveillance activity, approved, and endorsed by the Waco-McLennan County Public Health District. As such, this project met exclusion criteria for institutional review board approval at 45 CFR 46.102(e) and (l) for Baylor University researchers, staff, and volunteers. Participation of Waco Family Medicine researchers, staff, and volunteers for the present project was approved by the institutional review board at Ascension Providence Hospital and Medical Center of Waco, Texas.

### Materials

#### Clinical Risk Score for Severe COVID-19

In order to estimate each participant's approximate risk for severe COVID-19 disease, risk scores were calculated using information about demographic characteristics and pre-existing conditions previously shown to increase the odds of experiencing severe disease (CDC, 2020; Chidambaram et al., 2020; Petrilli et al., 2020). These included sex, smoking status, age, race/ethnicity, body mass index (BMI), and whether the participant reported ever being diagnosed with diabetes, cardiovascular disease, chronic obstructive pulmonary disease (COPD), kidney disease, liver disease, or cancer. Consistent with previous research examining how these factors contribute to COVID-19 outcomes (Petrilli et al., 2020), categorical variables were computed for age (19–44, 45–54, 55–64, 65–74, and >74 years old), and BMI (<25, 25–29.99, 30–39.99, >39.99).

Following methods used to develop clinical risk scores for other diseases and adverse medical events (Fong et al., 1999; Mehran et al., 2004; Sullivan et al., 2004; Callery et al., 2013; Zheutlin et al., 2019), each factor was weighted based on the strength of its association with severe COVID-19 disease found in previous studies. Specifically, weights were odds ratios (adjusted) for the effects of each factor derived from the results of logistic regression analyses reported in recently published meta-analyses and cohort studies (Chidambaram et al., 2020; Petrilli et al., 2020)^1^. Reference groups for each factor (e.g., those without diabetes, cardiovascular disease, etc.) were assigned a weight of 1. These weights were summed and divided by eleven (the number of factors included in the score), with higher quotients representing a greater estimated susceptibility to severe COVID-19 disease outcomes. For example:

$$\begin{array}{l} Clinical\;risk\;score=\frac{condition1^{*}odds\;ratio\;+condition2^{*}odds\;ratio\ldots +condition11^{*}odds\;ratio}{11} \end{array}$$

#### COVID-19 Threat Appraisal

To assess the extent to which participants believed they were vulnerable to COVID-19 disease, questions were asked about their perceived likelihood of becoming infected by the SARS-CoV-2 virus and, if infected, their likelihood of experiencing severe COVID-19 disease. Specifically, participants responded to the questions: “What do you consider to be your own probability of getting infected with COVID-19?” and “How severe would contracting COVID-19 be for you (how seriously ill do you think you will be)?” Participants responded using 7-point Likert-type scale (1 = very low; 7 = very high). A third question was also answered using the same scale: “How would you rate your knowledge level on how to prevent spread of COVID-19?” This latter item was included to control for participants' perceived knowledge about SARS-CoV-2 virus transmission when examining perceptions of risk.

#### Self-Isolation During Shelter-in-Place Order and Travel

Participants were asked about the frequency at which they left their homes during the Texas shelter-in-place order (March 13 through May 1, 2020). They were further asked approximately how many times in an average week they left their homes during that period for the following reasons: buying essential supplies (e.g., groceries or water), going to a friend's house, going to a gas station, going to a liquor store, picking up food from a restaurant, and going to a public park. Participants responded with a whole number for each activity.

Participants were also asked about their travel following March 13, 2020, providing information about dates and locations for all travel outside of their city of residence (up to five trips). The total number of trips made within Texas and outside of Texas were calculated separately. The latter number included both out of state and out of country travel, as only five participants reported trips outside of the United States following March 13.

#### Psychological Distress During the Pandemic

Participants completed three measures to estimate psychological distress during the pandemic. First, participants were asked to rate their agreement with two statements: “COVID-19 makes me feel helpless” and “COVID-19 makes me depressed” using 7-point Likert-type scale (1 = strongly disagree; 7 = strongly agree). Second, participants completed the short-form Perceived Stress Scale (PSS-4) (Herrero and Meneses, 2006). This scale assesses the frequency at which participants feel stressed and overwhelmed in the month prior to their participation date (α = 0.80).

#### Covariates

The following additional variables that may covary with perceived or actual COVID-19 risk, social distancing behavior and travel, and/or psychological distress during the pandemic were included in the analyses (see Tables 1–3 for additional details about these variables): general risk tolerance (i.e., “Are you generally someone who tends to take risks, or do you tend to avoid risks?”), education, self-rated knowledge about COVID-19, whether or not the participant had health insurance, number of cohabitants in households, whether or not the participant worked as a healthcare provider or first responder, average daily encounters with a co-worker or friend/family member within six feet without a face covering^2^, and whether the participant: (a) knew someone who had been diagnosed with COVID-19, (b) knew someone who had been hospitalized with COVID-19, (c) knew someone who died from COVID-19, (d) provided care for a COVID-19 patient, and (e) had been within six feet of someone who had been diagnosed with COVID-19.

**Table 1:** Basic demographic statistics.

**Characteristic**	***N* (*%*)**	**Mean (*SD*)**	**Median (range)**
**Total**	492 (100)		
**Age**	492 (100)	44.4 (14.25)	42 (19–87)
**Group**			
3	*180 (36.6)*		
4	*108 (22.0)*		
5	*100 (20.3)*		
6	*88 (17.9)*		
7	*16 (3.3)*		
**Sex/Gender**			
Female	309 *(62.8)*		
Male	183 *(37.2)*		
**Racial identification**			
American Indian or Alaskan Native	*1 (0.2)*		
Asian	6 *(1.2)*		
Black/American Indian	*12 (2.4)*		
Native Hawaiian or other Pacific Islander	*1 (0.2)*		
White	*430 (87.4)*		
More than one race	*31 (6.3)*		
Other/Missing	*11 (2.2)*		
**Hispanic, Latino, or of Spanish origin**			
Yes	*96 (19.5)*		
No	*395 (80.3)*		
**Currently employed**			
Yes	*413 (83.9)*		
No	*79 (16.1)*		
**Worked as healthcare provider or a first responder since December 2019**			
Yes	*177 (36.0)*		
No	*314 (63.8)*		
**Currently have health insurance**			
Yes	*460 (93.5)*		
No	*32 (6.5)*		
**Type of insurance**			
Medicare	*44 (8.9)*		
Private	*417 (84.6)*		
Other/Missing	*32 (6.5)*		
**Highest level of education**			
No high school or GED	*1 (0.2)*		
High school/GED	*12 (2.4)*		
Some college	*71 (14.4)*		
2-year degree (associate's)	*67 (13.6)*		
Professional certification	*31 (6.3)*		
4-year college degree	*136 (27.6)*		
Some grad school, no degree	*28 (5.7)*		
Master's degree	*91 (18.5)*		
Doctoral degree	*51 (10.4)*		
Other graduate degree	*4 (0.8)*		
**Cohabitants in household**	492 (100)	2.2 (1.63)	2 (0–10)
**Childhood SES–partial**	492 (100)	3.8 (1.52)	3.7 (1–7)
**Political affiliation**			
Democrat or lean democrat	*133 (27.0)*		
No lean	*137 (27.8)*		
Republican or lean republican	*219 (44.5)*		

### Data Analysis Plan

Descriptive statistics are displayed in Tables 2–3. All analyses were conducted using SPSS (v27) and MPlus (v8) statistical software. All *p* values were two-tailed and considered significant at the level *p* < 0.05. First, variables were examined for normality and the presence of outliers (i.e., three standard deviations above or below the mean). Data for frequency of travel and leaving home during the shelter-in-place order, as well as the risk score, were positively skewed. Accordingly, model parameters were estimated using robust maximum likelihood estimation in MPlus, an estimation method that is robust to non-normality (Yuan and Bentler, 2000). Additionally, data for 70 participants contained outliers for at least one variable. Models were tested both with and without these outlying data points included; any changes in the pattern or significance of results across these models are noted in the “Results” section (see also Table 4).

**Table 2:** Health descriptive statistics.

**Characteristic**	***N* (*%*)**	**Mean (*SD*)**	**Median (range)**
**BMI**	492 (100)	29 (6.42)	28.1 (16.1–56.5)
**Chronic infectious diseases**			
Yes	*4 (0.8)*		
No	*488 (99.2)*		
**Chronic non-infectious diseases or medical conditions**			
Yes	*216 (43.9)*		
No	*271 (55.1)*		
**COVID-19 disease related pre-existing conditions (≥1)**			
Yes	*205 (41.7)*		
No	*287 (58.3)*		
**Specific COVID-19 disease related pre-existing conditions (self-reported)**			
Age >65	*48 (9.8)*		
Any cardiovascular/heart condition, including COPD, congestive heart failure, and hypertension	*58 (11.8)*		
COPD	*1 (0.2)*		
Any chronic lung disease, including moderate or severe asthma	*27 (5.5)*		
Chronic kidney disease	*3 (0.6)*		
Liver disease	*2 (0.4)*		
Diabetes	*19 (3.9)*		
Obesity	*98 (19.9)*		
Having an immune deficiency, including HIV	*3 (0.6)*		
Receiving cancer treatment or other immune weakening medications including corticosteroids	*3 (0.6)*		
Smoking or vaping	*36 (7.3)*		
Living in a nursing home or long-term care facility	*0 (0.0)*		
**Influenza vaccine since September 2019**			
Yes	*336 (68.8)*		
No	*156 (31.7)*		
**Smoking and vaping**			
Smoking	*29 (5.9)*		
*Cigarettes per day*		*10 (5.74)*	*10 (0–30)*
Vaping	*10 (2.0)*		
*Puffs per day*		*72 (91.74)*	*26.5 (12–300)*
Neither	*452 (91.9)*		
**Smokeless tobacco or nicotine products use**			
Yes	*19 (3.9)*		
No	*473 (96.1)*		
**Hours per week spend on physical exercise**	**491 (99.8)**	**7.7 (24.43)**	**4 (0–500)**
**Diet**			
On a diet	103 (20.9)		
No special diet	373 (75.8)		
**COVID-19 disease risk score**	492 (100)	1.1 (0.1)	1 (1–1.7)

**Table 3:** Exposure, risk behavior, and risk perception.

**Characteristic**	***N* (*%*)**	**Mean (*SD*)**	**Median (range)**
**Contact within 6 feet per day with**			
Coworker or client/patron before March 13th	*467 (94.9)*	*22.5 (38.21)*	*10 (0–500)*
Friend or family member before March 13^*th*^	*465 (94.5)*	*13.8 (21.54)*	*8 (0–203)*
Coworker or client/patron between May 1st and May 18th/22nd	*467 (94.9)*	*6.6 (16.31)*	*1 (0–200)*
Friend or family member between May 1st and May 18th/22nd	*466 (94.7)*	*10.8 (20.58)*	*5 (0–200)*
Coworker or client/patron after May 18th/22nd	*467 (94.9)*	*5.9 (15.64)*	*1 (0–200)*
Friend or family member after May 18th/22nd	*466 (94.7)*	*10.4 (20.54)*	*4 (0–200)*
**Exposure to**			
Animal with confirmed or suspected COVID-19 disease			
*Yes*	*10 (2.0)*		
*No*	*481 (97.8)*		
Know someone who has been diagnosed with COVID-19 disease			
*Yes*	*335 (68.1)*		
*No*	*157 (31.9)*		
Know someone who has been hospitalized from COVID-19 disease			
*Yes*	*140 (28.5)*		
*No*	*352 (71.5)*		
Know someone who has passed away from COVID-19 disease			
*Yes*	*81 (16.5)*		
*No*	*410 (83.3)*		
Provide a care for a COVID-19 disease patient			
*Yes*	*88 (17.9)*		
*No*	*403 (81.9)*		
Be within 6 feet of anyone that has been diagnosed with COVID-19 disease			
*Yes*	*146 (29.7)*		
*No*	*346 (70.3)*		
**Wash hands with soap and water or use sanitizer**			
Before the pandemic	*491 (99.8)*	*9.9 (10.57)*	*6 (0–100)*
Since the pandemic	*491 (99.8)*	*17.5 (15.07)*	*12 (1–100)*
Change before-after	*491 (99.8)*	*7.6 (9.06)*	*5 (−15–97)*
**Left home between March 13th and May 1st**			
To buy essential supplies	*491 (99.8)*	*2.8 (4.78)*	*2 (0–60)*
To a friend's house	*488 (99.2)*	*0.7 (2.95)*	*0 (0–60)*
To a gas station	*491 (99.8)*	*1.9 (3.01)*	*1 (0–42)*
To a liquor store	*486 (98.8)*	*0.3 (0.84)*	*0 (0–10)*
Pick up food from a restaurant	*488 (99.2)*	*2.8 (4.53)*	*2 (0–56)*
To a public park	*490 (99.6)*	*1.2 (3.68)*	*0 (0–60)*
**Times traveled within Texas after March 13**	492 (100)	0.9 (1.3)	0.5 (0–5)
**Times traveled outside of Texas after March 13**	492 (100)	0.2 (0.5)	0.0 (0–3)
**Self-rated knowledge level on how to prevent spread of SARS-CoV-2 virus**	490 (99.6)	6.1 (0.95)	6 (1–7)
**Self-rated probability of getting infected with SARS-CoV-2 virus**	489 (99.4)	4.1 (1.59)	4 (1–7)
**Self-rated severity of contracting COVID-19 disease**	489 (99.4)	3.6 (1.52)	4 (1–7)
**Self-rated adherence to recommendations from authorities in the country to prevent spread of SARS-CoV-2 virus**	490 (99.6)	7 (1.67)	8 (1–8)
**Willingness to take a vaccine if available**	491 (99.8)	5.5 (1.98)	6 (1–7)
**Willingness to live up to restrictions, even if not formal anymore, if there is a surge in cases of COVID-19**	491 (99.8)	5.5 (1.64)	6 (1–7)
**Self-reported feeling helpless because of COVID-19 disease**	490 (99.6)	3.9 (2.26)	4 (1–8)
**Self-reported feeling depressed because of COVID-19 disease**	490 (99.6)	3.8 (2.3)	4 (1–8)
**Tend to take risk**	491 (99.8)	3.6 (1.74)	4 (1–7)
**Perceived Vulnerability to Disease Perceived Infectability subscale (PVD-DP)-partial**	492 (100)	3.1 (1.26)	3 (1–7)
**Perceived Vulnerability to Disease Germ Aversion subscale (PVD-DP)-partial**	492 (100)	5.1 (1.14)	5 (1.3–7)
**Perceived stress scale-partial**	492 (100)	2.4 (0.73)	2.3 (1–4.8)
**Three domains of disgust scale-partial**	491 (99.8)	4.6 (1.34)	4.7 (1–7)

**Table 4:** Results of statistical models.

	**Unstandardized beta coefficient (*****SE*****)**		
**Dependent variable**	**Primary model**	**Covariates included**	**Outliers removed**
**Risk perception**			
Likelihood of infection	−2.63 (0.84)^**^	−1.80 (0.87)^*^	−2.93 (0.97)^**^
Likelihood of severe illness if infected	3.79 (0.64)^***^	3.38 (0.70)^***^	4.10 (0.95)^***^
**Behavior during shelter-in-place order**			
Buy supplies	−0.48 (2.19)	−0.50 (2.28)	0.11 (1.19)
Visit friend	7.14 (6.74)	7.25 (6.87)	−0.20 (0.87)
Gas station	−1.74 (1.11)	−1.50 (1.12)	−0.71 (1.24)
Liquor store	0.16 (0.48)	0.25 (0.47)	0.001 (0.35)
Pick up food from restaurant	−3.92 (1.67)^*^	−4.12 (1.73)^*^	−2.57 (1.87)
Public park	−1.85 (0.82)^*^	−2.07 (0.85)^*^	−1.78 (1.46)
**Travel during pandemic**			
Within State	1.83 (0.57)^**^	1.84 (0.58)^**^	1.71 (0.70)^*^
Outside of State	−0.98 (0.94)	−1.43 (0.99)	−1.01 (1.98)
**Psychological distress during pandemic**			
Perceived stress	−1.21 (0.32)^***^	−1.17 (0.34)^**^	−1.26 (0.49)^*^
Feelings of depression	−3.37 (1.04)^**^	−4.27 (1.09)^***^	−3.28 (1.44)^*^
Feelings of helplessness	−3.60 (1.09)^**^	−5.27 (1.12)^***^	−3.72 (1.50)^*^

*SE, standard error*.

* *p < 0.05*,

** *p < 0.01*,

*** *p < 0.001*.

To examine the relationship between estimated risk for severe COVID-19 disease and the outcomes of perceived risk, behavior during the shelter-in-place order, and psychological distress, we simultaneously regressed each dependent variable on risk scores in a multivariate model. The variables measuring the frequency of travel outside of participants' home city were modeled as count data (with a high frequency of zeroes) and parameter estimates were generated using negative binomial regression (Gardner et al., 1995). Because the variables assessing the average number of times participants left their home (but stayed within the city) included non-integers (e.g., 0.5 times per week on average), these data were not modeled as count data (i.e., standard linear regression parameter estimation was used). This model was tested a second time controlling for covariates (see above for full list). Zero-order correlations between the estimated COVID-19 severity risk score and all covariates are displayed in Table 5. Given the non-normality of the risk measure, Spearman rank-order correlation procedure was used to estimate coefficients and significance values.

**Table 5:** Spearman rank correlations between COVID-19 disease risk score and covariates.

**Variable**	**1**	**2**	**3**	**4**	**5**	**6**	**7**	**8**	**9**	**10**	**11**	**12**
1. COVID-19 disease severity risk score	-											
2. Risk tolerance	−0.02	-										
3. Education	−0.07	0.03	-									
4. Self-rated knowledge about COVID-19 disease	0.02	−0.03	0.14^***^	-								
5. Has health insurance	0.06	−0.03	0.14^***^	0.03	-							
6. Number of cohabitants	−0.27^***^	0.04	−0.04	−0.10^*^	0.01	-						
7. Healthcare worker or first responder	−0.19^***^	0.03	−0.03	0.18^***^	0.06	0.07	-					
8. Interactions without face coverings	−0.11^*^	0.19^***^	−0.09^*^	−0.09^*^	−0.03	0.26^***^	0.11^*^	-				
9. Know someone diagnosed with COVID-19 disease	0.03	−0.02	−0.01	−0.03	0.01	−0.01	0.01	−0.04	-			
10. Know someone hospitalized with COVID-19 disease	0.08	0.06	0.10^*^	0.03	−0.09^*^	−0.04	−0.004	−0.03	0.39^***^	-		
11. Know someone who died from COVID-19 disease	0.07	−0.08	0.004	0.07	−0.07	−0.02	0.05	−0.08	0.22^***^	0.49^***^	-	
12. Provided care for someone with COVID-19 disease	−0.17^***^	0.03	0.05	0.20^***^	0.06	−0.01	0.49^***^	0.07	0.15^***^	0.11^*^	0.08	-
13. Within six feet of someone with COVID-19 disease	−0.10^*^	0.11^*^	−0.05	0.15^***^	−0.03	−0.04	0.26^***^	0.09	0.27^***^	0.19^***^	0.13^**^	0.65^***^

* *p < 0.05*,

** *p < 0.01*,

*** *p < 0.001*.

## Results

Characteristics of the sample are shown in Table 1. A total of 495 participants completed the behavioral survey (women: 311, men: 183, other: 1, *M*_age_ = 44.43, *SD*_age_ = 14.28). Three participants provided incomplete data for the variables needed to calculate the risk score and were thus excluded from analyses.

### COVID-19 Threat Appraisal

Unstandardized beta coefficients and standard errors for parameters in all models are displayed in Table 4. Results of the regression analysis revealed that as participants' clinical risk score for severe COVID-19 disease increased, their perceived risk of experiencing severe illness if infected also increased (*b* = 3.79, *SE* = 0.64, *t* = 5.96, *p* < 0.001). In contrast, higher clinical risk scores for severe COVID-19 disease predicted lower perceived risk of becoming infected with SARS-CoV-2 virus (*b* = −2.63, *SE* = 0.84, *t* = −3.12, *p* = 0.002). In other words, while individuals with a high clinical risk score seemed to acknowledge their elevated likelihood of experiencing severe COVID-19 if infected, they actually reported lower perceived risk of becoming infected in the first place. Removing outliers did not change the pattern or significance of these results (likelihood of severe illness: *p* < 0.001; likelihood of infection: *p* = 0.002), nor did controlling for covariates (likelihood of severe illness: *p* < 0.001; likelihood of infection: *p* = 0.037).

### Self-Isolation During the Shelter-in-Place Order and Travel

Regarding travel within the participants' city of residence during the shelter-in-place order, higher clinical risk scores for severe COVID-19 disease predicted a lower frequency of going to a park (*b* = −1.85, *SE* = 0.82, *t* = −2.27, *p* = 0.023) and picking up food from a restaurant (*b* = −3.29, *SE* = 1.67, *t* = −2.35, *p* = 0.019). However, clinical risk scores were not significantly related to frequency of leaving the home to purchase supplies (*b* = −0.48, *SE* = 2.19 *t* = −0.22, *p* = 0.825), visiting a friend (*b* = 7.14, *SE* = 6.74, *t* = 1.06, *p* = 0.299), going to a gas station (*b* = −1.74, *SE* = 1.11, *t* = −1.57, *p* = 0.118), or going to a liquor store (*b* = 0.16, *SE* = 0.48, *t* = 0.33, *p* = 0.741).

While these results did not change when controlling for covariates (park: *p* = 0.015, restaurant: *p* = 0.017, supplies: *p* = 0.827, friend: *p* = 0.291, gas station: *p* = 0.182, liquor store: *p* = 0.598), all effects—including going to a park or restaurant—became non-significant when outliers were removed (park: *p* = 0.222, restaurant: *p* = 0.170, supplies: *p* = 0.930, friend: *p* = 0.822, gas station: *p* = 0.567, liquor store: *p* = 0.999). The change in statistical significance that occurred after outlying values were excluded may indicate that participants who reported very high frequencies of these activities did so in error. Specifically, it is possible that certain participants reported the total number of times they engaged in each activity between March 13 and May 1 instead of the average number of times per week.

While higher clinical risk scores did not significantly predict frequency of travel outside of the state of Texas (*b* = −0.98, *SE* = 0.94, *t* = −1.04, *p* = 0.299), those with higher scores reported a greater number of trips within Texas than those with lower scores (*b* = 1.83, *SE* = 0.57, *t* = 3.20, *p* = 0.001). The pattern and significance of these results did not change when controlling for covariates (outside of state: *p* = 0.145; within state: *p* = 0.001), nor did they change when outliers were excluded (outside of state: *p* = 0.609; within state: *p* = 0.015).

In sum, these findings suggest that individuals at a high risk for severe COVID-19 do not generally behave differently than those at low risk. Specifically, clinical risk scores were not reliably associated with participants' frequency of leaving their homes during the shelter-in-place order, nor were they related to out-of-state travel. Unexpectedly, participants with higher clinical risk scores actually reported traveling more often outside of their resident city, but within their home state of Texas, than those with lower risk scores.

### Psychological Distress During the Pandemic

Higher clinical risk scores for severe COVID-19 disease were associated with lower perceived stress (i.e., PSS-4 scale) (*b* = −1.21, *SE* = 0.32, *t* = −3.79, *p* < 0.001), feeling less depressed by the pandemic (*b* = −3.37, *SE* = 1.042, *t* = −3.24, *p* = 0.001), and feeling less helpless in response to the pandemic (*b* = −3.60, *SE* = 1.09, *t* = −3.31, *p* = 0.001). In other words, despite their increased likelihood of severe COVID-19 if infected, individuals with higher clinical risk scores appear to experience less psychological distress than those with lower scores. The pattern and significance of these results did not change when controlling for covariates (stress: *p* = 0.001; depressed: *p* < 0.001; helpless: *p* < 0.001), nor did they change when outliers were removed (stress: *p* = 0.010; depressed: *p* = 0.023; helpless: *p* = 0.013).

## Discussion

### Evolution, Unrealistic Optimism, and COVID-19

Unrealistic optimism is a common human feature. Despite posing the potential cost of promoting risky behavior in the face of uncertain outcomes (Weinstein, 1982; Michailova and Schmidt, 2016; Karl et al., 2020), unrealistic optimism may also provide a number of psychological and health benefits (Johnson and Fowler, 2011; Kleiman et al., 2017; Rozanski et al., 2019). The decreased worry associated with unrealistic optimism may improve mental well-being of some individuals during the COVID-19 pandemic, which is associated with mental health and sleep disturbances (Pappa et al., 2020; Pfefferbaum and North, 2020), and overconfidence may increase productivity through increasing morale and persistence (Johnson and Fowler, 2011). It is also possible that overconfidence may decrease productivity by setting unrealistic goals and failing which can lead to psychological and financial struggles (Makridakis and Moleskis, 2015). Given the possible benefits of overconfidence, it has been proposed that positively-biased affect, beliefs, and attitudes were favored by natural selection (Johnson and Fowler, 2011). In line with this hypothesis, behavior consistent with optimism biases have been observed in a variety of non-human animal species (Harding et al., 2004; Matheson et al., 2008).

Despite some hypothesized psychological benefits discussed above, unrealistic optimism can be dangerous due to improper assessment of hazardous situations, and inferring that optimism bias has evolutionary origins does not mean that unrealistic optimism is an “optimal” strategy in every situation. This is especially true when individuals are faced with a novel source (or scale) of risk that was not present in the environments under which optimism biases may have evolved. For example, unrealistic optimism about one's probability of becoming infected with the SARS-CoV-2 virus or of developing severe COVID-19 disease may be maladaptive, leading to behaviors that increase one's odds of exposure. This could be especially harmful for those with high risk of developing severe COVID-19 disease if infected. In the case of COVID-19, a disease with a high percentage of asymptomatic cases (Cheng et al., 2020; Huff and Singh, 2020), a long contagious period before showing symptoms (Tindale et al., 2020), it is possible that for some people it can be challenging to recognize the risk of becoming infected with the virus (Varella et al., 2021).

Age and comorbidities are strong indicators of hospital admission and, to a lesser degree, mortality among patients with COVID-19 disease (Petrilli et al., 2020). Men over the age of 65 and smokers are among the highest at risk for adverse outcomes of COVID-19 disease (Zheng et al., 2020). Other factors that may influence COVID-19 disease outcomes include comorbidities like hypertension, diabetes, respiratory diseases, or cardiovascular diseases (Albitar et al., 2020; Li et al., 2020; Zheng et al., 2020). More specifically, chronic obstructive pulmonary disease (COPD), cerebrovascular disease, and cardiovascular disease are associated with severe COVID-19 disease (Sole et al., 2020). People with cancer also experience higher severity of COVID-19 disease in conjunction with diagnostic and therapy delays (ElGohary et al., 2020). However, it must be noted that most, if not all, systematic reviews and meta-analyses at this time are skewed toward populations in China; thus, the exact risk resulting from a pre-existing condition may differ across populations.

While age and other comorbidities have been clearly linked to adverse outcomes of COVID-19 (Petrilli et al., 2020), many high-risk individuals continue to disregard public health guidelines and recommendations. Though concern for the novel coronavirus is persistent across borders, risk perception may also be culturally biased. For example, countries that abide by strict cultural norms reported almost five times fewer cases of COVID-19 and almost eight times lower number of deaths, suggesting a strong influence of this factor on recommendation compliance and risk perception (Gelfand et al., 2021). Additionally, factors such as personal experience, prosocial and individual values, trust in government (Dryhurst et al., 2020), life history strategy (Corpuz et al., 2020), and sex (Luoto and Varella, 2021) all influence risk perception. Individual differences in disease avoidance motivation also likely play important mediating or moderating roles in relationships between actual risk, perceived risk, and behavior (Makhanova and Shepherd, 2020). Future research is needed to examine how each of these factors influence the pattern of results found in the current research.

Risk perception correlates significantly with use of preventative health behaviors for COVID-19 in ten different countries (Dryhurst et al., 2020). In the United States, people reported a higher perceived risk of COVID-19 disease compared to other current health threats (Zhong et al., 2020). Despite this, many people still do not sufficiently understand SARS-CoV-2 virus transmission and COVID-19 disease prevention (Zhong et al., 2020). An individual's perception of their own risk to a threat impacts their health behaviors (Ferrer and Klein, 2015), and unrealistic optimism, especially among high-risk individuals, may be partly responsible for the avoidance of necessary preventative measures during the current pandemic (Dolinski et al., 2020). With the threat of the SARS-CoV-2 virus continually growing, understanding risk perception and subsequent behavioral outcomes regarding COVID-19 are essential to public health.

### Perceived Risk and Compliance With State Law and Public Health Recommendations

The present project examined whether one's calculated clinical risk for severe COVID-19 disease (based on the aforementioned demographic and clinical characteristics) was related to risk perception, behavior, and psychological distress during the current pandemic. The data reveal that, while individuals with a higher clinical risk score accurately report greater perceived risk for severe illness, they actually perceive a lower risk of being infected with SARS-CoV-2 virus (relative to those with fewer or no pre-existing conditions). Although counterintuitive, the finding is consistent with the wide body of research on unrealistic optimism in the context of health risk management (Sharot et al., 2011; Shepperd et al., 2015; Jefferson et al., 2017). In particular, the current results provide further support for research demonstrating that unrealistic optimism about the likelihood of experiencing adverse health outcomes is greater for those with a higher number of risk factors (Karl et al., 2020). It must be noted here that not all studies support the cost-benefit basis of the evolution of unrealistic bias, and in some cases, it can be a potentially costly cognitive bias (Jefferson, 2017).

Within the present dataset, there is not reliable evidence that those at higher risk for severe COVID-19 disease traveled less during and after the shelter-in-place order (March 13 through May 1) than those with lower risk. That is, clinical risk scores for severe COVID-19 disease were not significantly related to individuals' frequency of leaving their homes to engage in activities like visiting friends or going to the grocery store. While higher clinical risk for severe COVID-19 disease initially predicted fewer trips to the park and picking up food from restaurants, these effects were not statistically significant after outliers were removed. Moreover, results revealed that higher risk for severe COVID-19 disease was actually associated with more trips outside of the participants' resident city (but within the state). In other words, individuals with a higher number of pre-existing conditions did not appear to exercise more caution than those with fewer conditions, despite their elevated risk for morbidity and mortality from COVID-19. While these results may be surprising at face value, they lend further support for the prediction that those at a high risk for severe COVID-19 are unrealistically optimistic about their likelihood of infection. It is important to consider that if those at a high risk for severe COVID-19 disease are willing to break shelter-in-place orders and other public health recommendations, it is likely that other individuals without pre-existing conditions are also willing to disregard safety recommendations. Many people unfortunately choose to disregard public health recommendations during the current pandemic for various reasons (e.g., personal, social, political, etc.), and unrealistic optimism may be contributing to poor decision making in many of these individuals. On the other side, one study has found that islandic, Croatian men scored higher on perceptions of infectability during the COVID-19 pandemic than before, while women did not, and the authors hypothesize that it may reflect the objectively higher risk of COVID-19 (Hromatko et al., 2021). This seems to suggest that at least some amount of increased personal risk for infection is acknowledged by higher-risk individuals during the current pandemic.

Results of the present study also revealed that higher clinical risk scores are associated with less reported stress, and fewer feelings of depression and helplessness during the pandemic. One plausible explanation is that, in believing that they are less likely to contract the SARS-CoV-2 virus, individuals with higher risk for severe COVID-19 disease experience fewer negative emotions. While these results are consistent with research finding that unrealistic optimism acts as a psychological buffer from stress (Taylor et al., 1992; Taylor and Brown, 1994; Makridakis and Moleskis, 2015), that those with a higher disease risk actually reported less distress than those with a lower disease risk is unexpected. In other words, this pattern of results suggests that unrealistic optimism in the face of serious COVID-19 risk goes beyond merely assuaging negative emotions. Instead, distress may be suppressed by optimism to a level below even what those without pre-existing conditions report. Another possible explanation is that those with higher clinical risk scores are less likely to have experienced the distress associated with a friend or loved one developing severe COVID-19 disease. However, risk scores were not significantly related to whether or not participants knew someone who was hospitalized or passed away from COVID-19 disease (see Table 5).

It is worth noting that, within the present dataset, those at a higher risk for severe COVID-19 disease had smaller households and were less likely to report being a first responder/healthcare worker or to have cared for someone with COVID-19 disease (see Table 5). Higher clinical risk scores were also negatively correlated with the number of maskless interactions reported. It is therefore possible that, in this dataset, those with a higher risk for severe COVID-19 disease truly are less likely to become infected with the SARS-CoV-2 virus. In other words, do these individuals exhibit unrealistic optimism or do they realistically evaluate their risk of becoming infected? Although this cannot be determined definitively with the current cross-sectional data, there does not appear to be strong evidence for the latter possibility. Participants in this dataset with a higher disease risk score did not generally behave differently than those with a lower disease risk score during the shelter-in-place order, and they were actually more likely to travel outside of their resident city. Additionally, neither the pattern nor significance of the relationship between risk scores and perceived likelihood of infection changed after controlling for possible occupational exposure, number of cohabitants, and other factors that may influence infection probability. Nonetheless, it remains possible that an unmeasured covariate confounds the relationship between clinical risk scores and risk perception; this is a limitation of the current study.

Another potential limitation is that it is difficult to determine with the current data which risk factors for severe COVID-19, specifically, drive relationships between clinical risk scores and risk perceptions. In the present study, the three most common risk factors were age, male sex, and high BMI. However, each of these are likely to have unique effects on risk perception and behavior. For example, while one might be less mobile and risk averse in advanced age, male sex is conversely associated with greater engagement in risky behaviors during the pandemic, higher mobility, and lower adherence to preventative activities (Galasso et al., 2020; Luoto and Varella, 2021). Moreover, it is also difficult to discern which factor is driving COVID-19 risk, *per se*, because age is a common predictor of nearly all of the more potent risk factors, like diabetes, cardiovascular disease, etc. For this reason and others, it is important to exercise caution when thinking about cumulative risk in such a reductionist sense. That is, it is unlikely that one's total risk is truly just the sum of independent predictors. Accordingly, that we only apply an additive model of clinical risk without secondary validation in separate samples is a limitation of the current study. Future research using larger sample sizes might explore multiplicative and non-linear effects, as well as redundancy between risk factors. Meta-analyses are well-suited for this purpose.

A final limitation includes how unrealistic optimism was assessed. In the current study, risk perception was deemed unrealistic because those with higher clinical risk scores reported being less likely to become infected than those with lower scores, despite little evidence of this being true. It should be noted, however, that optimism biases are typically measured in a comparative fashion. That is, respondents are asked about their risk relative to others' (Weinstein, 1982; Ferrer et al., 2012; Shepperd et al., 2015). Future research is needed to determine whether a similar pattern of results would be found using a more commonly employed method of assessing unrealistic optimism.

### Future Directions

The ecological fallacy prevents the results from the current dataset to be generalized to other populations within and outside of the United States. Almost 500 asymptomatic volunteers were selected from over 1,000 applicants of McLennan County, TX, residents based on the following self-reported risk factors: if they were a frontline worker or healthcare provider, if they were an essential employee, if they broke shelter-in-place orders to attend religious and other services/activities in person, or if they have followed all shelter-in-place and public health orders/recommendations. The dataset does not represent completely random selection from among the county residents and does not accurately reflect the percentage distribution of those above/below the poverty line (e.g., only 6.5% did not have health insurance) or minority status (e.g., only 19.5% of the dataset included Hispanic and Latina/Latino members). The dataset is also over-represented by individuals with above-average education who therefore likely have above-average concern about the current pandemic, although it is unlikely that differences in perceived risk are attributed to lack of education given the public health messaging about the current pandemic. However, the primary compensation from the present study was a free IgG antibody test for the SARS-CoV-2 virus, and those uninterested in knowing their serological status to the virus are less likely to be represented in the present dataset. Future work would ideally (but with great difficulty) include completely randomly selected community members resulting in a more diverse dataset.

One particularly interesting area for future research includes cross-cultural comparison of risk perception and its influence on preventative behaviors in relation to COVID-19 disease. Emerging infectious diseases (EIDs) have been a persistent global problem, and Asia has historically been an epicenter for many of these outbreaks. A considerable amount of effort has been put into surveillance and prevention in countries like India, where infectious disease outbreaks have been common (Mukherjee, 2017). In the United States and other high-income countries, the primary causes of mortality include non-communicable diseases (NCDs). Because of this trend, the United States healthcare infrastructure is primarily designed to manage the high prevalence of NCDs rather than EIDs. This may have exacerbated the lack of preparedness of the United States for the SARS-CoV-2 virus pandemic at the country, state, and individual levels (Katzmarzyk et al., 2020). Furthermore, cultural differences in tightness-looseness (Gelfand et al., 2021), as well as potential regional biological differences reflected in motivation to avoid infectious diseases (Skolnick and Dzokoto, 2013; Gassen et al., 2018; Cepon-Robins et al., 2021; Krams et al., 2021), may also influence certain countries' tendencies to invest heavily in pandemic preparedness.

In sum, the results of the current research provide partial evidence for a miscalibration between one's actual risk for severe COVID-19 disease, perceptions of risk, and behaviors that mitigate that risk. This study may lay the groundwork for future research to examine, in more detail, how unrealistically optimistic perceptions about infection likelihood and severity contribute to the spread of SARS-CoV-2 virus, particularly for those with pre-existing conditions.

## Data Availability Statement

The raw data supporting the conclusions of this article will be made available by the authors, without undue reservation.

## Data Sharing

De-identified data can be made available to researchers upon reasonable request.

## Ethics Statement

The studies involving human participants were reviewed and approved by Ascension Providence Hospital, Waco, TX. The patients/participants provided their written informed consent to participate in this study.

## Author Contributions

MM and EB conceived the Waco COVID Survey and implemented it with SW. MM wrote the survey, designed the study, and obtained the funding. EB designed and managed the websites. TN, JG, and MM managed the enrollment. JG and TN lead the data collection. MM and JG conceived the paper. TN and JG conducted the statistical analyses. AH contributed to data collection and manuscript preparation. JG, TN, AH, and MM wrote the manuscript.

## Conflict of Interest

The authors declare that the research was conducted in the absence of any commercial or financial relationships that could be construed as a potential conflict of interest.

## Footnotes

^1^Odds ratios for sex, smoking status, diabetes, cardiovascular disease, COPD, kidney disease, liver disease, and cancer were obtained from Chidambaram et al., 2020 (Table 1). Odds ratios for age, race/ethnicity, and BMI were obtained from Petrilli et al., 2020 (Table 3).

^2^Mean composite (α = 0.78) of average number of encounters each day with (a) friend/family member prior to March 13, (b) co-worker prior to March 13, (c) friend/family member between May 1 and May 18^th^/22^nd^, (d) co-worker between May 1 and May 18^th^/22^nd^, (e) friend/family member after May 18^th^/22^nd^, and (f) co-worker after May 18^th^/22^nd^.
