# Supplementary material for: Unrealistic Optimism and Risk for COVID-19 Disease
Source: Front Psychol. 2021 Jun 4;12:647461. doi: 10.3389/fpsyg.2021.647461 (PMC8212979; doi:10.3389/fpsyg.2021.647461)

Supplementary Materials for

Unrealistic Optimism and Risk for COVID-19 Disease

Jeffrey Gassen^1†^, Tomasz J. Nowak^1†^, Alexandria D. Henderson^1^, Sally P. Weaver^2^, Erich J. Baker^3^, Michael P. Muehlenbein^1*^

^1^ Department of Anthropology, Baylor University, Waco, Texas, United States

^2^ Waco Family Medicine, Waco, Texas, United States

^3^ Department of Computer Science, Baylor University, Waco, Texas, United States

^†^ These authors have contributed equally to this work and share first authorship.

***Correspondence:**

Michael P. Muehlenbein
[Michael_muehlenbein@baylor.edu](mailto:Michael_muehlenbein@baylor.edu)

**Included in file**

Supplementary Figures S1-S13

*Figure S1*. Perceived likelihood of infection plotted against clinical risk scores with outlying values included (top) and without these values included (bottom).


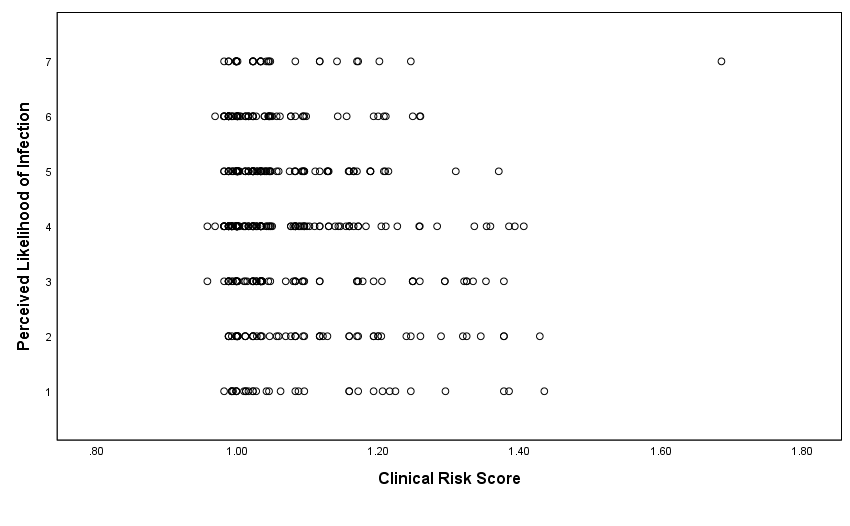


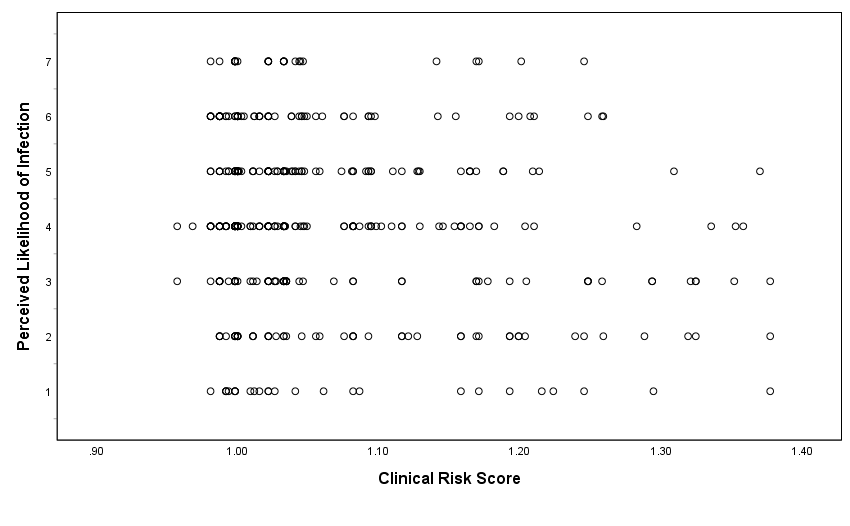


*Figure S2*. Perceived severity of illness if infected plotted against clinical risk scores with outlying values included (top) and without these values included (bottom).


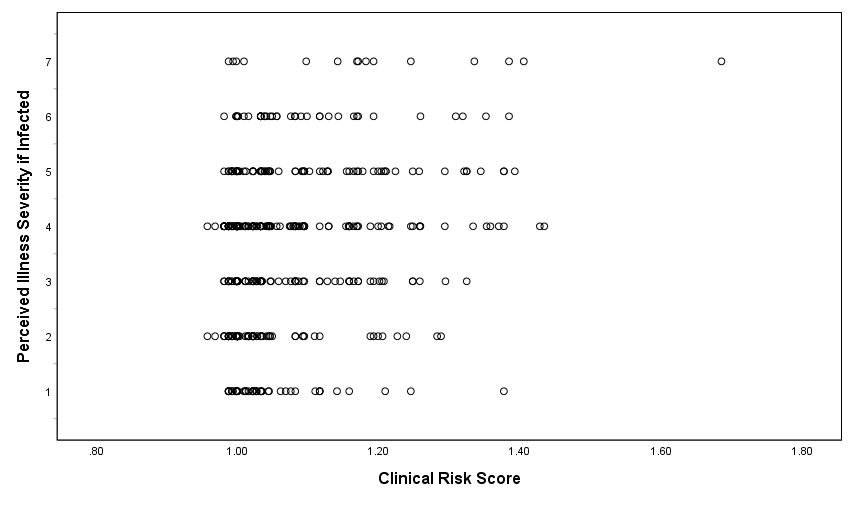


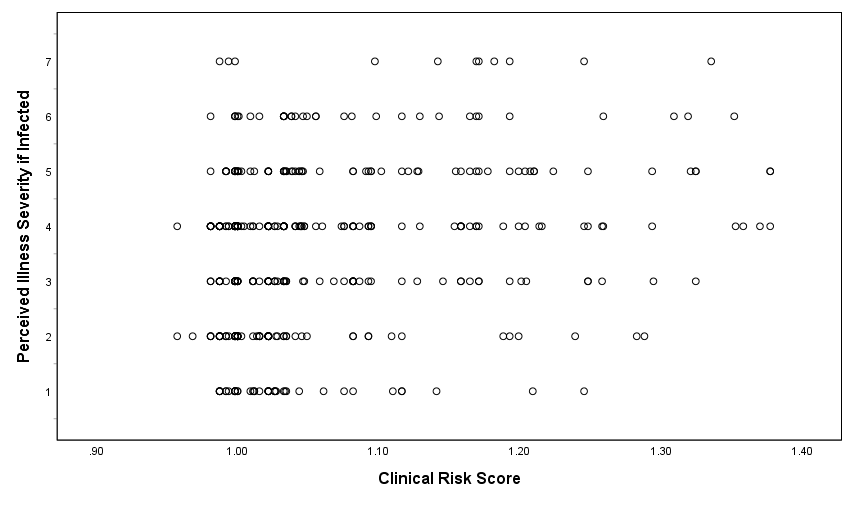


*Figure S3*. Average number of times per week left home to buy essential supplies plotted against clinical risk scores with outlying values included (top) and without these values included (bottom).


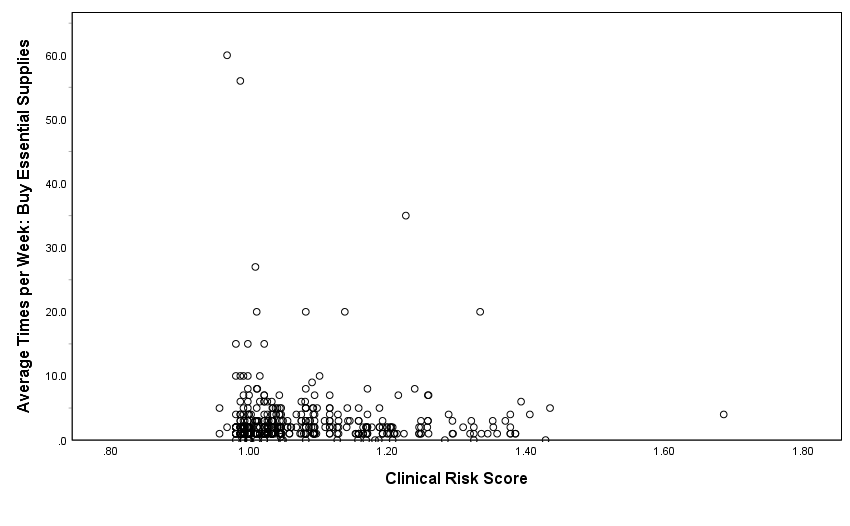


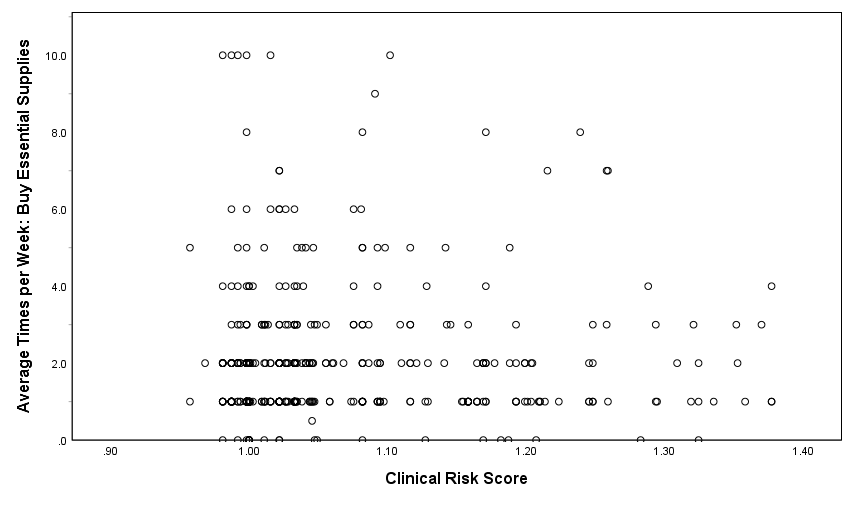


*Figure S4*. Average number of times per week visited a friend plotted against clinical risk scores with outlying values included (top) and without these values included (bottom).


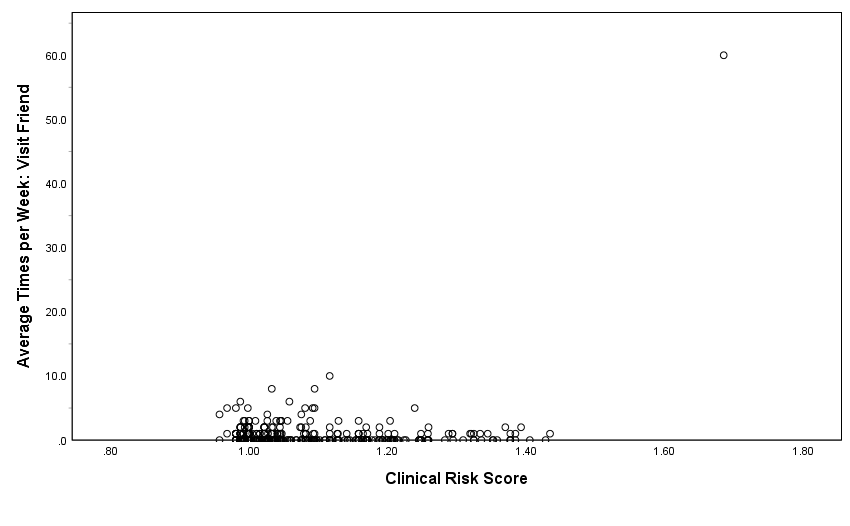


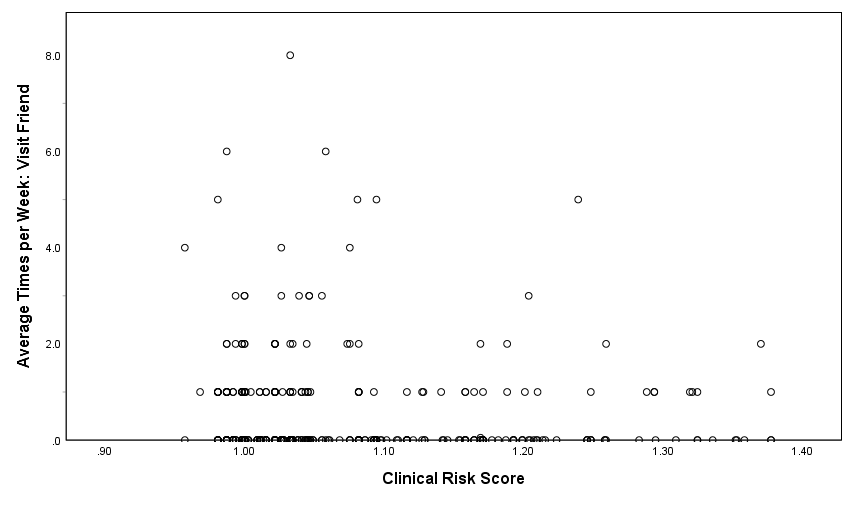


*Figure S5*. Average number of times per week left home to go to gas station plotted against clinical risk scores with outlying values included (top) and without these values included (bottom).


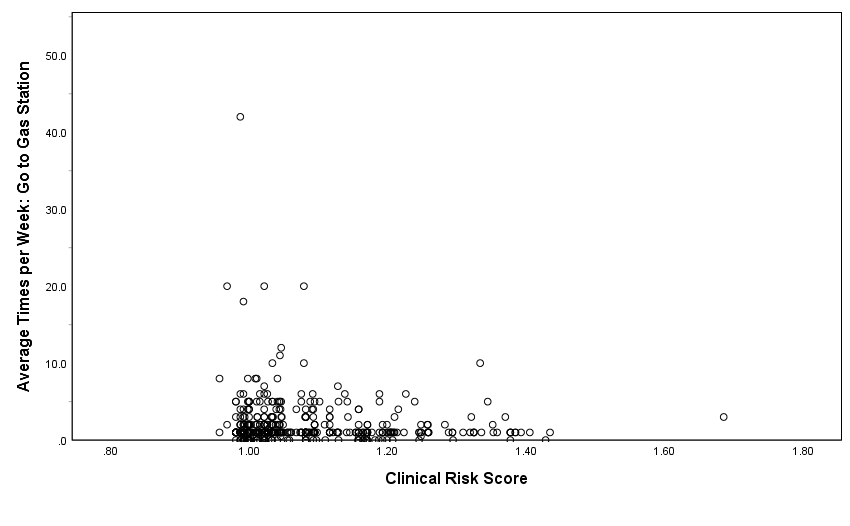


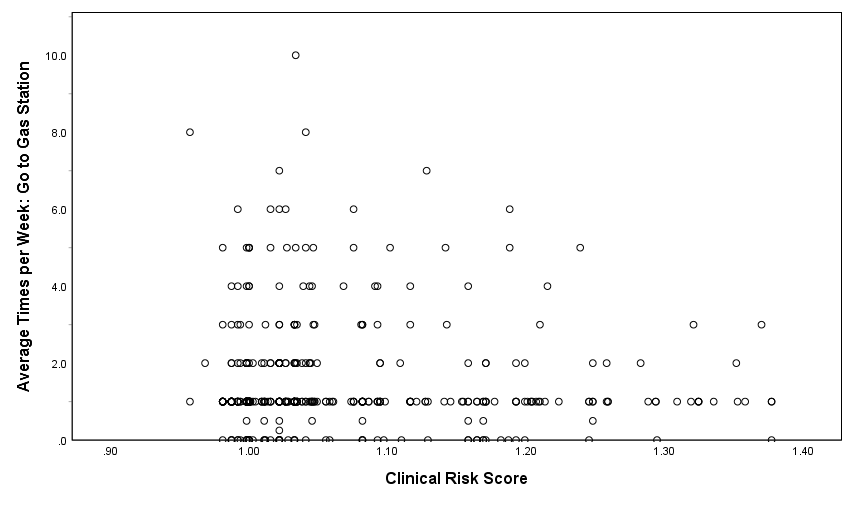


*Figure S6*. Average number of times per week left home to go to liquor store plotted against clinical risk scores with outlying values included (top) and without these values included (bottom).


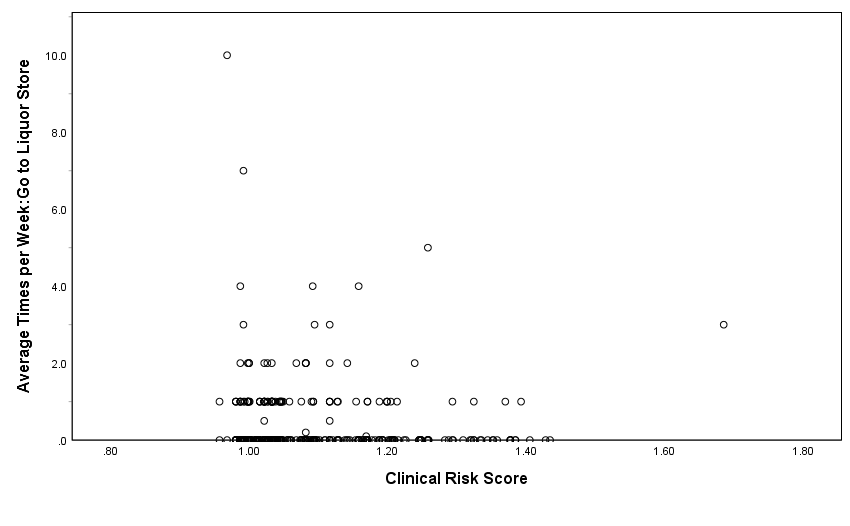


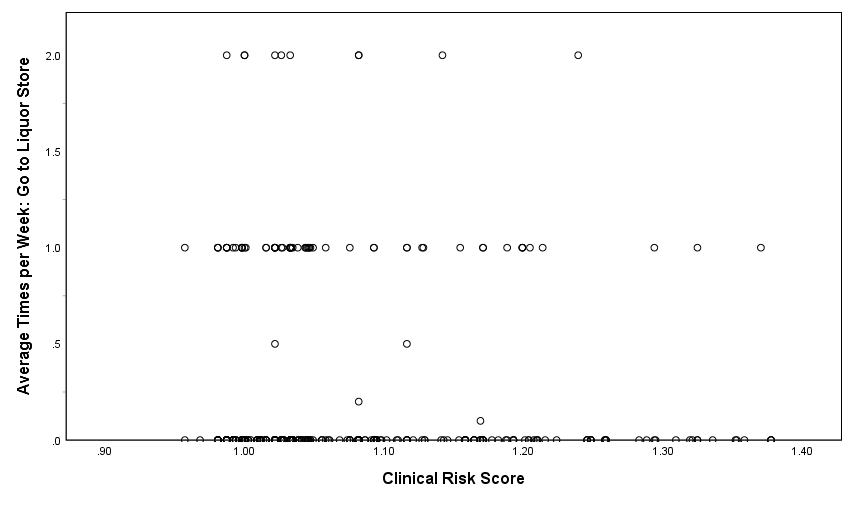


*Figure S7*. Average number of times per week left home to pick up food from restaurant plotted against clinical risk scores with outlying values included (top) and without these values included (bottom).


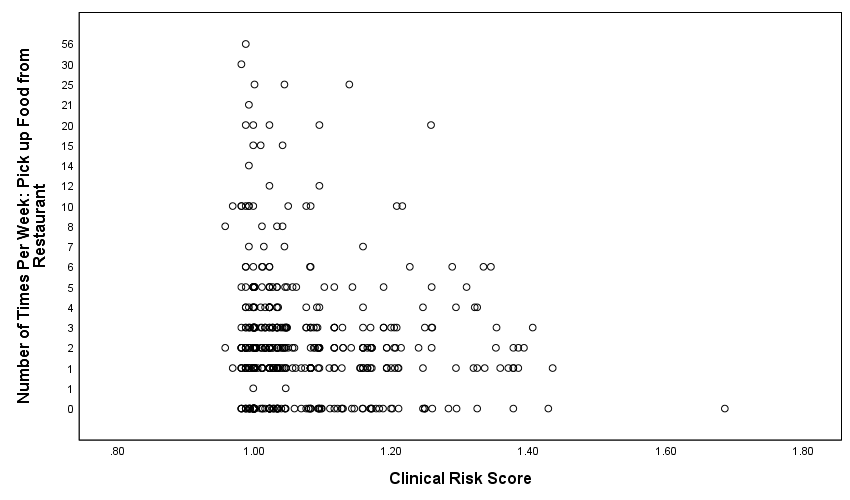


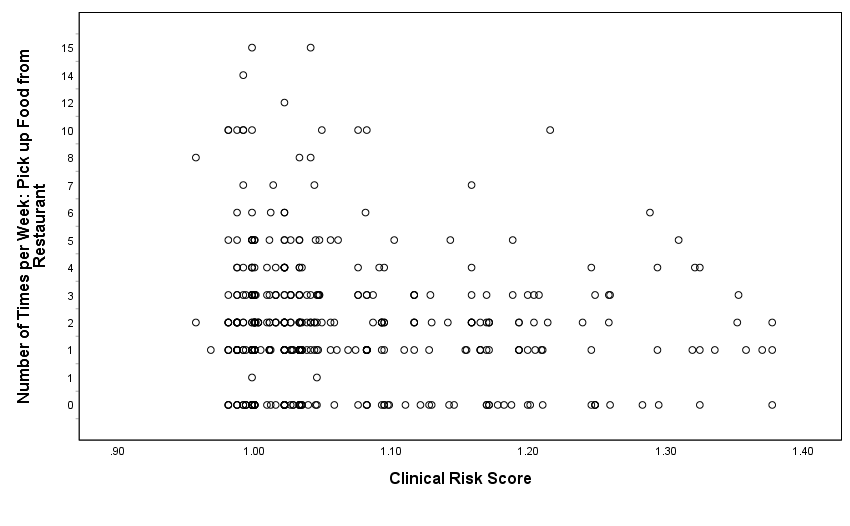


*Figure S8*. Average number of times per week went to a park plotted against clinical risk scores with outlying values included (top) and without these values included (bottom).


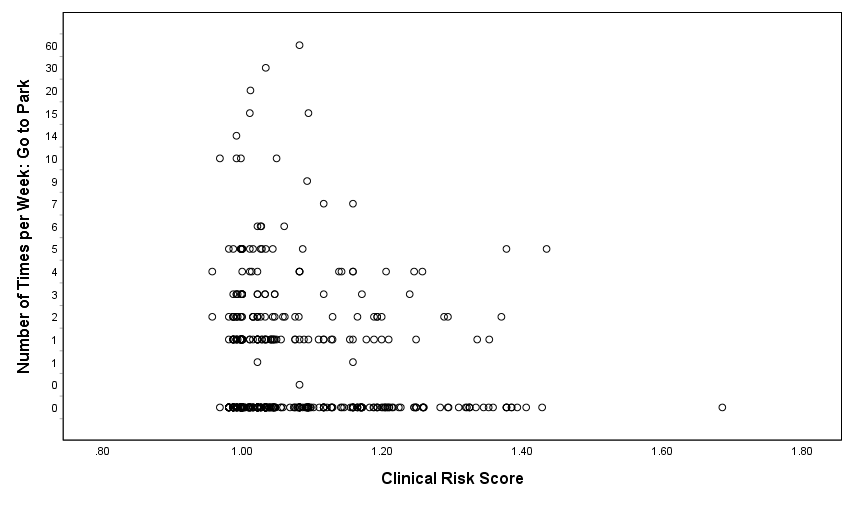


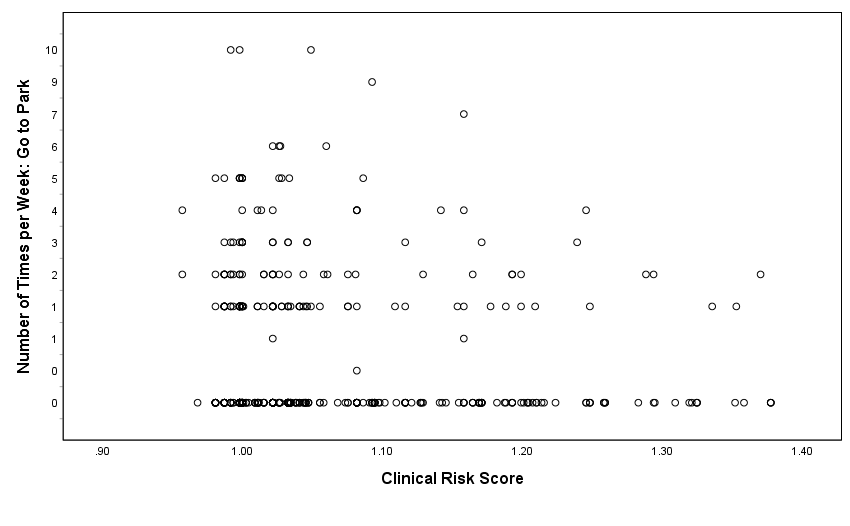


*Figure S9*. Number of times traveled within the state plotted against clinical risk scores with outlying values included (top) and without these values included (bottom).


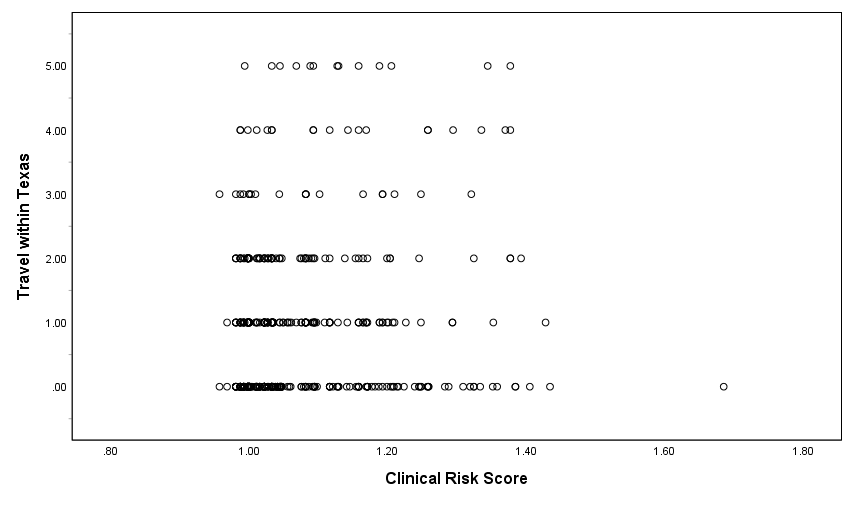


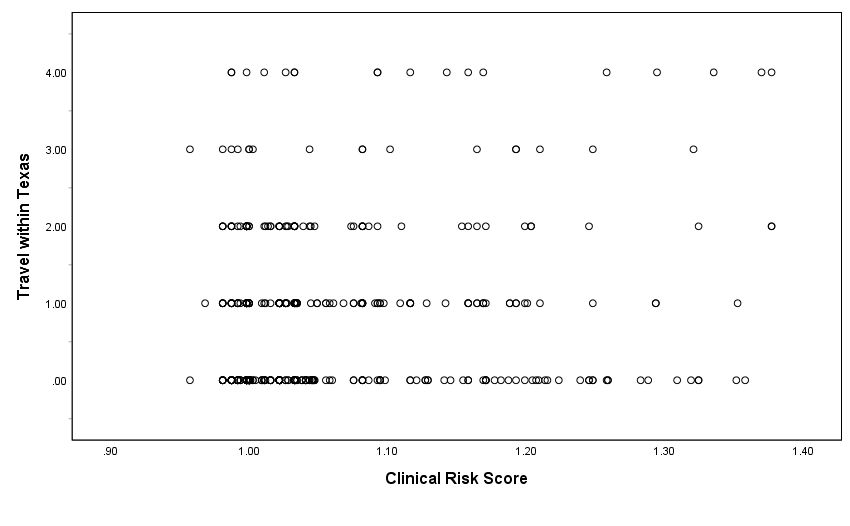


*Figure S10*. Number of times traveled outside of the state plotted against clinical risk scores with outlying values included (top) and without these values included (bottom).


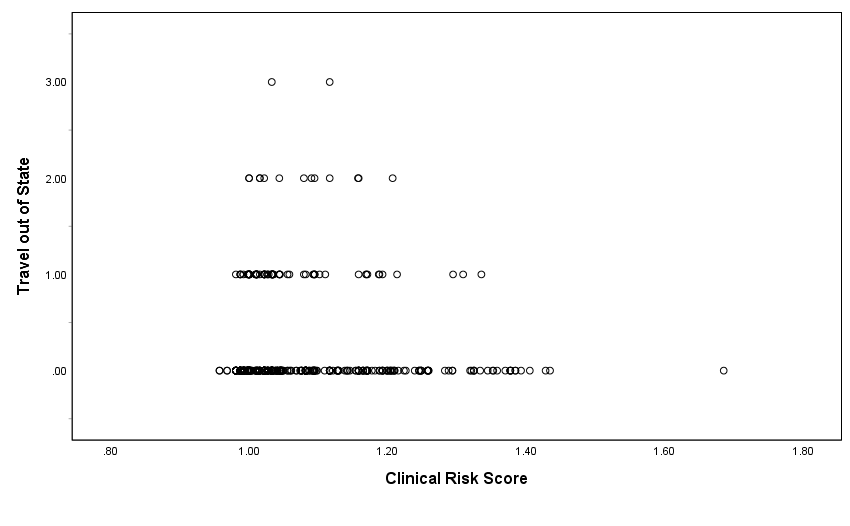


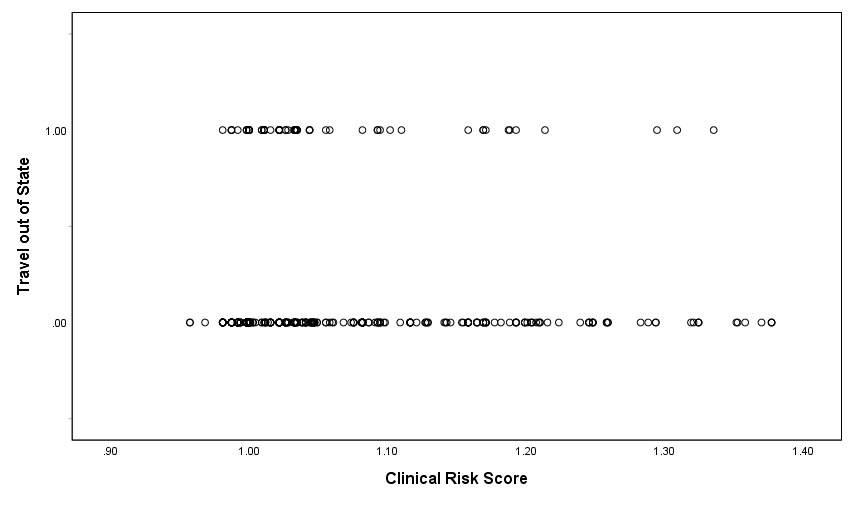


*Figure S11*. Perceived Stress Scale scores plotted against clinical risk scores with outlying values included (top) and without these values included (bottom).


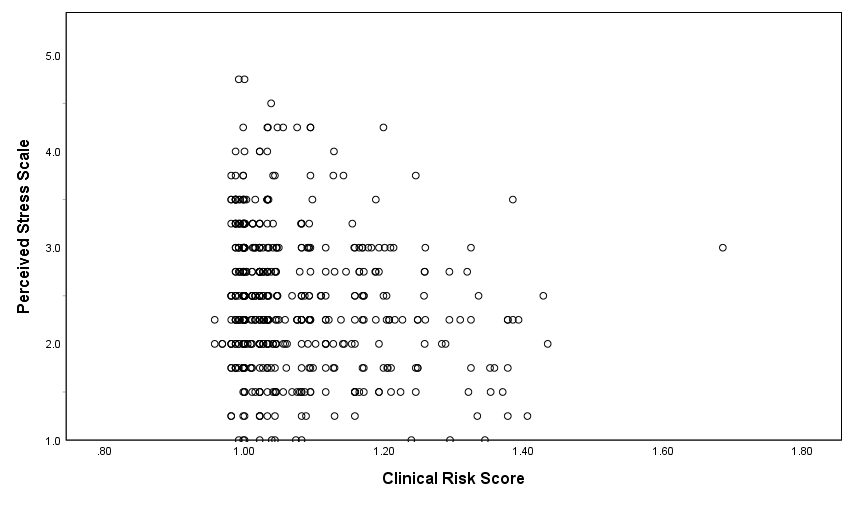


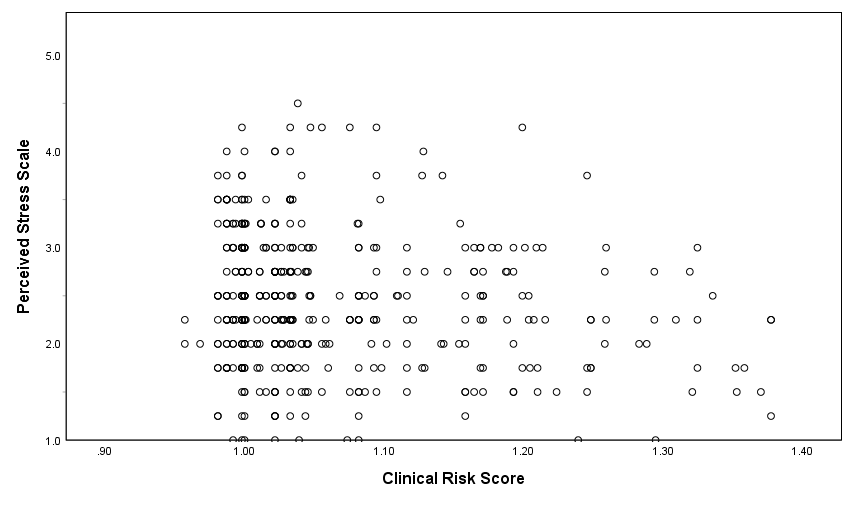


*Figure S12*. Feelings of helplessness plotted against clinical risk scores with outlying values included (top) and without these values included (bottom).


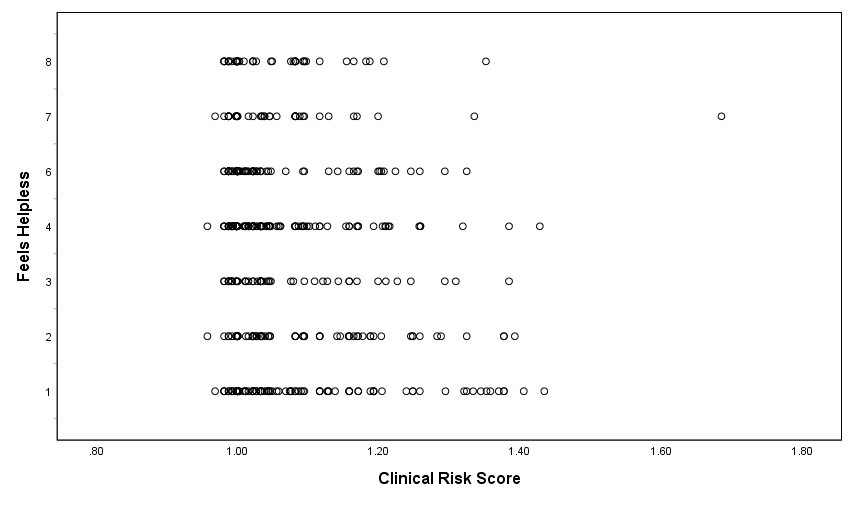


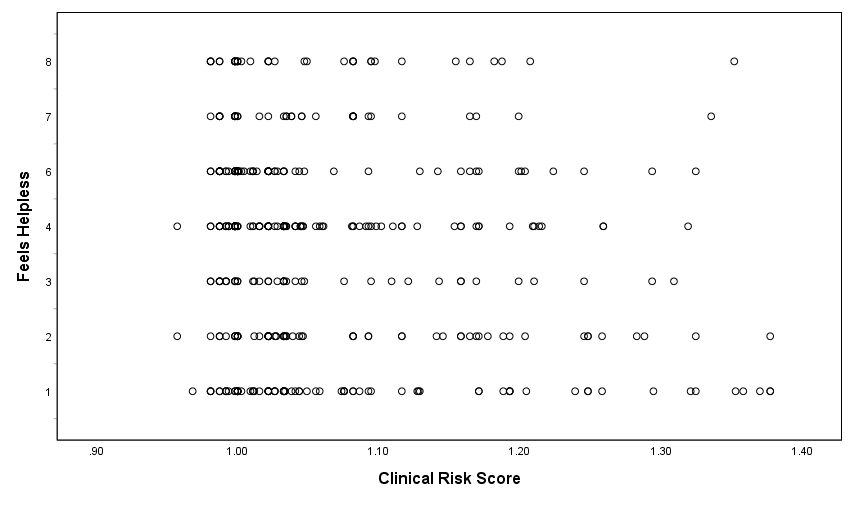


*Figure S13*. Feelings of depression plotted against clinical risk scores with outlying values included (top) and without these values included (bottom).


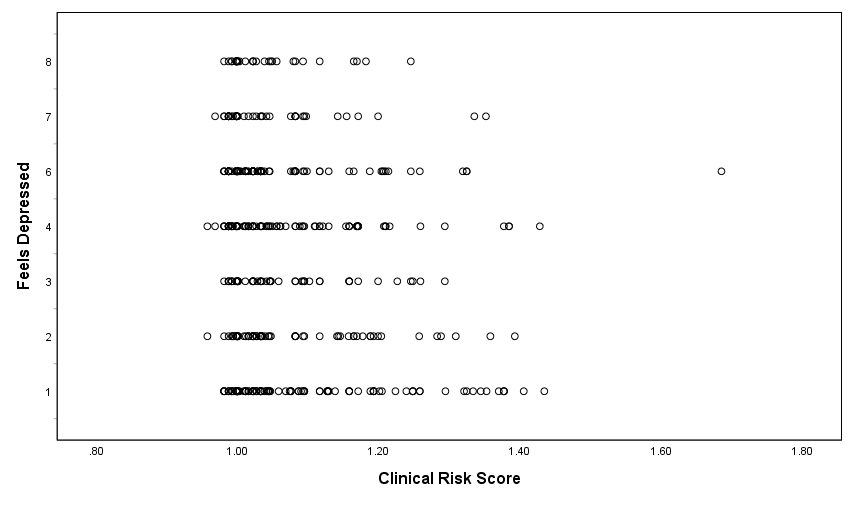


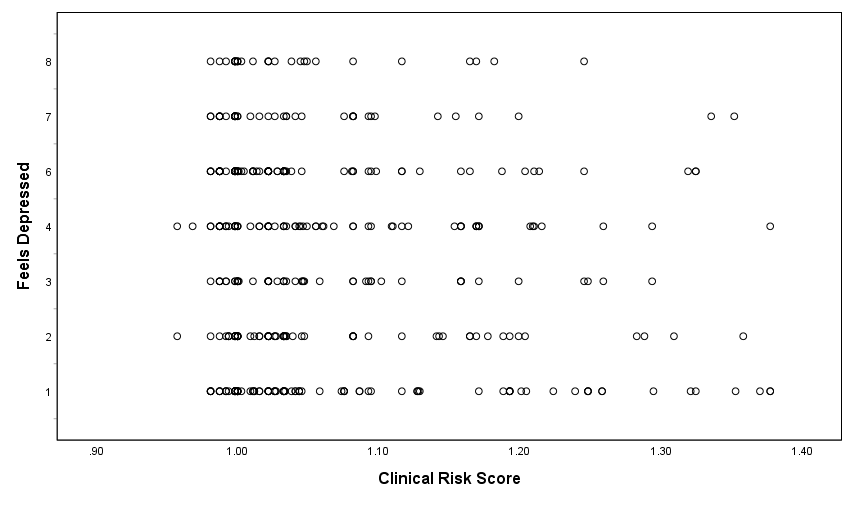

Supplement: Supplementary file 1 [file Data_Sheet_1.docx]
